# Supplementary material for: A Cross-Sectional Study on Disparities in Unmet Need among Refugees and Asylum Seekers in Thailand in 2019
Source: Int J Environ Res Public Health. 2021 Apr 8;18(8):3901. doi: 10.3390/ijerph18083901 (PMC8068097; doi:10.3390/ijerph18083901)
Supplement: Supplementary file 1 [file ijerph-18-03901-s001.pdf]

```
. **Conduct logistic regression for OP umnet need**
. logistic Reg_no_treatment i.New_country i.New_sex i.New_age ///
> i.Divided_hh_income i.New_past_sick i.New_chronic_disease

Logistic regression                             Number of obs   =        178
                                                LR chi2(8)      =      57.21
                                                Prob > chi2     =     0.0000
Log likelihood = -94.221733                     Pseudo R2       =     0.2329
```

Reg_no_treatment	Odds Ratio	Std. Err.	z	P>|z|	[95% Conf.	Interval]
New_country						
ARAB	14.68327	9.880823	3.99	0.000	3.926656	54.90633
OTHERS	2.691453	1.046859	2.55	0.011	1.255759	5.768561
New_sex						
Female	.955993	.3473193	-0.12	0.901	.4690368	1.948509
New_age						
More than 15 years but not over than 60 years	1.712135	.6569654	1.40	0.161	.8070912	3.632065
Over than 60 years	2.979117	4.112764	0.79	0.429	.1990571	44.58591
Divided_hh_income						
Income<=45707	3.490746	2.151798	2.03	0.043	1.042829	11.68486
New_past_sick						
Yes	4.321192	1.67455	3.78	0.000	2.021835	9.235523
New_chronic_disease						
Yes	1.95132	.9788446	1.33	0.183	.7300267	5.21577
_cons	.0407785	.0306445	-4.26	0.000	.009349	.1778682

```
. logit Reg_no_treatment i.New_country i.New_sex i.New_age ///
> i.Divided_hh_income i.New_past_sick i.New_chronic_disease

Iteration 0:   log likelihood = -122.82907
Iteration 1:   log likelihood = -94.462989
Iteration 2:   log likelihood = -94.222278
Iteration 3:   log likelihood = -94.221733
Iteration 4:   log likelihood = -94.221733

Logistic regression                             Number of obs   =        178
                                                LR chi2(8)      =      57.21
                                                Prob > chi2     =     0.0000
Log likelihood = -94.221733                     Pseudo R2       =     0.2329
```

Reg_no_treatment	Coef.	Std. Err.	z	P>|z|	[95% Conf.	Interval]
New_country						
ARAB	2.686708	.6729308	3.99	0.000	1.367788	4.005629
OTHERS	.9900814	.3889568	2.55	0.011	.2277401	1.752423
New_sex						
Female	-.0450047	.3633074	-0.12	0.901	-.7570741	.6670646
New_age						
More than 15 years but not over than 60 years	.5377414	.3837111	1.40	0.161	-.2143186	1.289801
Over than 60 years	1.091627	1.380531	0.79	0.429	-1.614164	3.797418
Divided_hh_income						
Income<=45707	1.250116	.6164291	2.03	0.043	.0419368	2.458294
New_past_sick						
Yes	1.463531	.3875204	3.78	0.000	.7040053	2.223057
New_chronic_disease						
Yes	.6685063	.5016319	1.33	0.183	-.3146742	1.651687
_cons	-3.199601	.7514877	-4.26	0.000	-4.67249	-1.726712

.

```
. **Then check probability that should be line between 0 and 1***
. predict predLOGISTIC
(option pr assumed; Pr(Reg_no_treatment))
(5 missing values generated)

. summ predLOGISTIC
```

Variable	Obs	Mean	Std. Dev.	Min	Max
predLOGISTIC	178	.5393258	.2673401	.0375212	.9813098

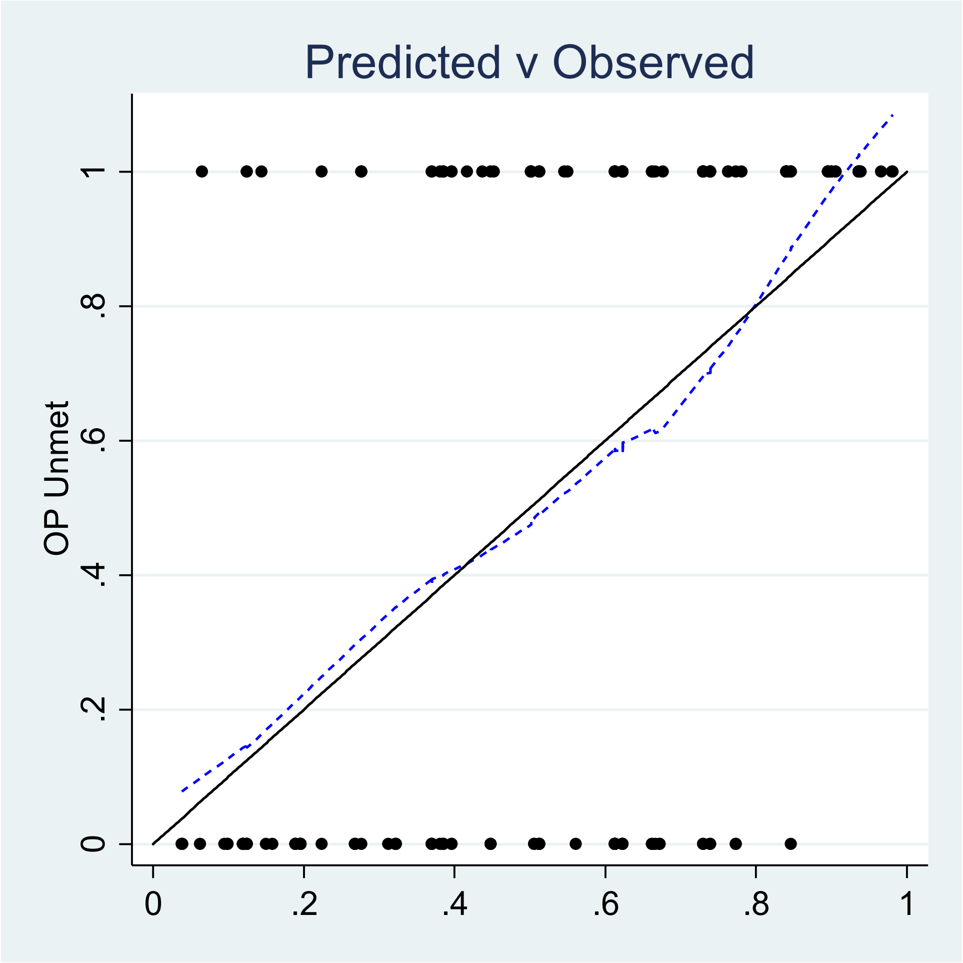

```
. **Check linear probability model**
. regress Reg_no_treatment i.New_country i.New_sex i.New_age ///
> i.Divided_hh_income i.New_past_sick i.New_chronic_disease

      Source |       SS       df       MS              Number of obs =     178
-------------+------------------------------           F(  8,   169) =    8.19
       Model |  12.3514827     8  1.54393534           Prob > F      =  0.0000
    Residual |  31.8732364   169  .188599032           R-squared     =  0.2793
-------------+------------------------------           Adj R-squared =  0.2452
       Total |  44.2247191   177   .24985717           Root MSE      =  .43428

----------------------------------------------------------------------------------------------------------
                                Reg_no_treatment |      Coef.   Std. Err.      t    P>|t|     [95% Conf. Interval]
-------------------------------------------------+--------------------------------------------------------
                                     New_country |
                                           ARAB  |   .4682442    .102677     4.56   0.000     .2655495    .6709389
                                         OTHERS  |   .1969934   .0736035     2.68   0.008     .0516927    .3422941
                                                 |
                                         New_sex |
                                         Female  |  -.0101872   .0668601    -0.15   0.879    -.1421758    .1218014
                                                 |
                                         New_age |
More than 15 years but not over than 60 years    |   .0902524   .0704178     1.28   0.202    -.0487594    .2292643
                              Over than 60 years |   .1068284   .2067557     0.52   0.606     -.301328    .5149848
                                                 |
                               Divided_hh_income |
                                  Income<=45707  |   .2164364   .1046446     2.07   0.040     .0098574    .4230155
                                                 |
                                   New_past_sick |
                                            Yes  |   .2840757   .0715875     3.97   0.000     .1427547    .4253967
                                                 |
                             New_chronic_disease |
                                            Yes  |   .1387457   .0932971     1.49   0.139    -.0454322    .3229236
                                           _cons |  -.0837571   .1144761    -0.73   0.465    -.3097444    .1422302
----------------------------------------------------------------------------------------------------------

. **Predict the probability of linear model**
. predict predLPM
(option xb assumed; fitted values)
(5 missing values generated)

. summ predLPM

    Variable |       Obs        Mean    Std. Dev.       Min        Max
-------------+--------------------------------------------------------
     predLPM |       178    .5393258    .2641636  -.0939443   1.130573
```

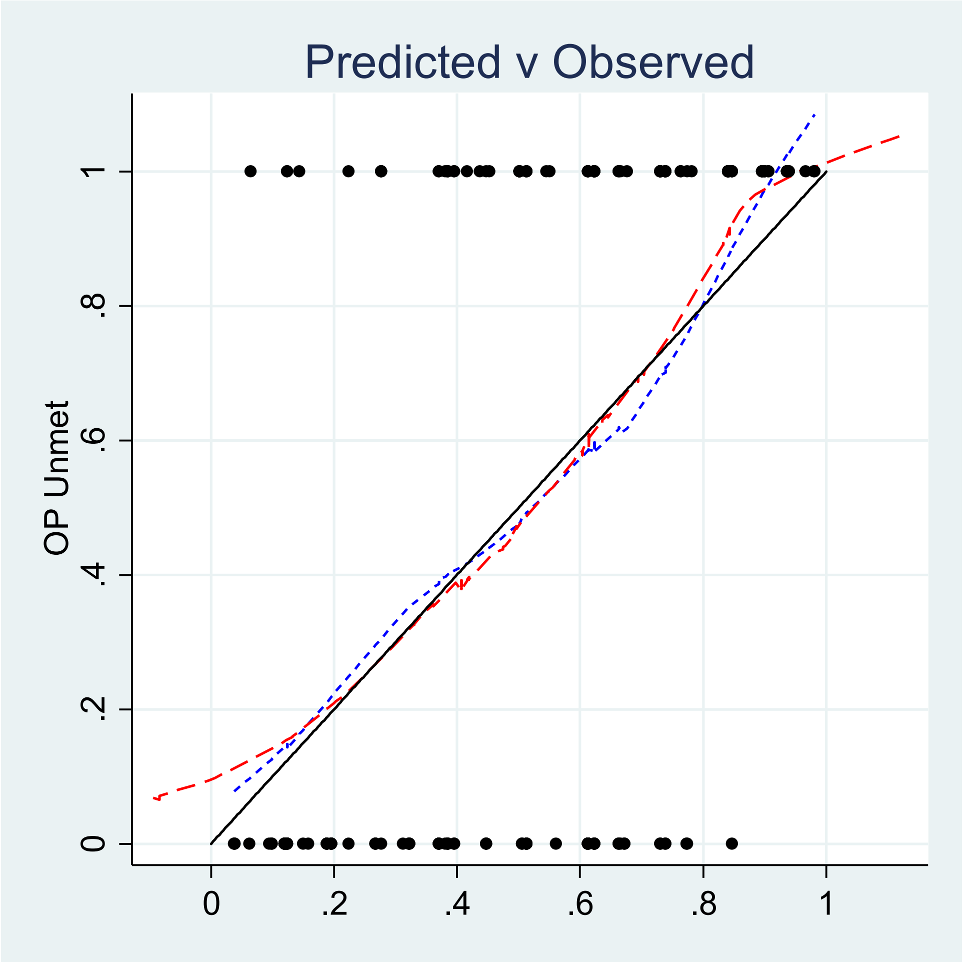
Predicted v Observed
OP Unmet
0
.2
.4
.6
.8
1
0
.2
.4
.6
.8
1

```
Logistic model for Reg_no_treatment, goodness-of-fit test

  (Table collapsed on quantiles of estimated probabilities)

       number of observations =       179
             number of groups =         8
      Hosmer-Lemeshow chi2(6) =        10.24
                  Prob > chi2 =         0.1150

. estat gof, group(10)

Logistic model for Reg_no_treatment, goodness-of-fit test

  (Table collapsed on quantiles of estimated probabilities)

       number of observations =       179
             number of groups =        10
      Hosmer-Lemeshow chi2(8) =         9.56
                  Prob > chi2 =         0.2972

. estat gof, group(20)

Logistic model for Reg_no_treatment, goodness-of-fit test

  (Table collapsed on quantiles of estimated probabilities)
  (There are only 14 distinct quantiles because of ties)

       number of observations =       179
             number of groups =        14
     Hosmer-Lemeshow chi2(12) =        13.38
                  Prob > chi2 =         0.3417

. estat gof, group(30)

Logistic model for Reg_no_treatment, goodness-of-fit test

  (Table collapsed on quantiles of estimated probabilities)
  (There are only 15 distinct quantiles because of ties)

       number of observations =       179
             number of groups =        15
     Hosmer-Lemeshow chi2(13) =        13.44
                  Prob > chi2 =         0.4144

.
end of do-file
```

```
. do "C:\Users\Watinee\AppData\Local\Temp\STD00000000.tmp"

. **Conduct logistic regression for IP umnet need**
. logistic Reg_no_admit i.New_country i.New_sex i.New_age ///
> i.Divided_hh_income i.New_past_sick i.New_chronic_disease

Logistic regression                               Number of obs   =        172
                                                  LR chi2(8)      =      37.80
                                                  Prob > chi2     =     0.0000
Log likelihood = -83.873532                       Pseudo R2       =     0.1839

------------------------------------------------------------------------------------------------------------------
                                   Reg_no_admit | Odds Ratio   Std. Err.      z    P>|z|     [95% Conf. Interval]
------------------------------------------------+-----------------------------------------------------------------
                                    New_country |
                                          ARAB  |   12.93422   7.874273     4.20   0.000     3.922239    42.65269
                                        OTHERS  |    2.23776   1.055119     1.71   0.088     .8881087    5.638464
                                                |
                                        New_sex |
                                        Female  |   1.341427    .530545     0.74   0.458     .6178886    2.912219
                                                |
                                        New_age |
More than 15 years but not over than 60 years  |   1.067697    .452857     0.15   0.877     .4649608    2.451771
                            Over than 60 years  |    .454485   .5784961    -0.62   0.536     .0375024     5.50783
                                                |
                              Divided_hh_income |
                                 Income<=45707  |   .7243369   .4767044    -0.49   0.624     .1994084    2.631103
                                                |
                                  New_past_sick |
                                           Yes  |   3.488072   1.703107     2.56   0.011     1.339592    9.082349
                                                |
                            New_chronic_disease |
                                           Yes  |   2.840068   1.426245     2.08   0.038     1.061369    7.599604
                                         _cons  |   .0560475   .0445077    -3.63   0.000     .0118198    .2657675
------------------------------------------------------------------------------------------------------------------

. logit Reg_no_admit i.New_country i.New_sex i.New_age ///
> i.Divided_hh_income i.New_past_sick i.New_chronic_disease

Iteration 0:   log likelihood = -102.77118
Iteration 1:   log likelihood = -84.936208
Iteration 2:   log likelihood = -83.879466
Iteration 3:   log likelihood = -83.873533
Iteration 4:   log likelihood = -83.873532

Logistic regression                               Number of obs   =        172
                                                  LR chi2(8)      =      37.80
                                                  Prob > chi2     =     0.0000
Log likelihood = -83.873532                       Pseudo R2       =     0.1839

------------------------------------------------------------------------------------------------------------------
                                   Reg_no_admit |      Coef.   Std. Err.      z    P>|z|     [95% Conf. Interval]
------------------------------------------------+-----------------------------------------------------------------
                                    New_country |
                                          ARAB  |   2.559877   .6087937     4.20   0.000     1.366663     3.75309
                                        OTHERS  |   .8054752   .4715068     1.71   0.088    -.1186612    1.729612
                                                |
                                        New_sex |
                                        Female  |   .2937341   .3955079     0.74   0.458    -.4814471    1.068915
                                                |
                                        New_age |
More than 15 years but not over than 60 years  |   .0655042   .4241437     0.15   0.877    -.7658021    .8968105
                            Over than 60 years  |  -.7885904   1.272861    -0.62   0.536    -3.283352    1.706171
                                                |
                              Divided_hh_income |
                                 Income<=45707  |  -.3224986   .6581252    -0.49   0.624      -1.6124    .9674031
                                                |
                                  New_past_sick |
                                           Yes  |   1.249349    .488266     2.56   0.011     .2923655    2.206333
                                                |
                            New_chronic_disease |
                                           Yes  |   1.043828   .5021868     2.08   0.038     .0595599    2.028096
                                         _cons  |  -2.881556   .7941076    -3.63   0.000    -4.437978   -1.325133
------------------------------------------------------------------------------------------------------------------

.
end of do-file
```

```
. **Then check probability that should be lined between 0 and 1***
. predict predLOGISTIC
(option pr assumed; Pr(Reg_no_admit))
(5 missing values generated)

. summ predLOGISTIC

    Variable |       Obs        Mean    Std. Dev.       Min        Max
-------------+--------------------------------------------------------
predLOGISTIC |       178    .2848995    .2070495   .0390134   .8816593

.
end of do-file
```

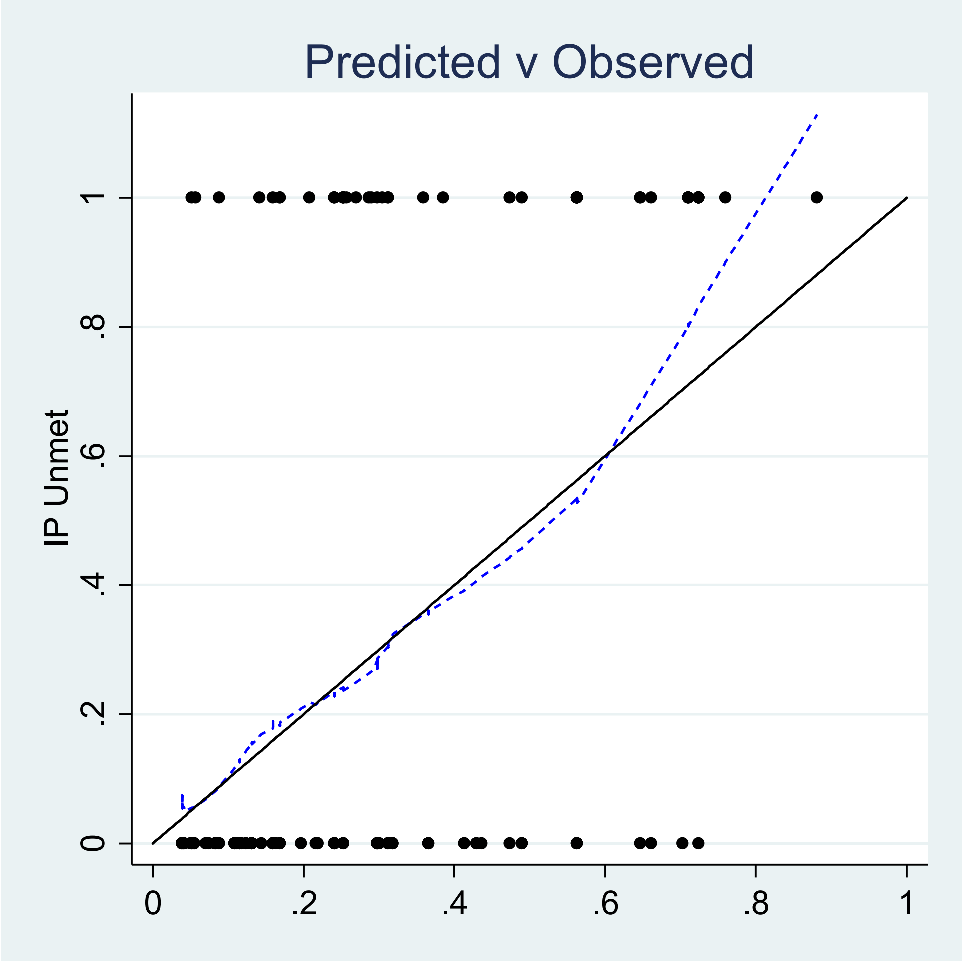

```
. **Check linear probability model**
. regress Reg_no_admit i.New_country i.New_sex i.New_age ///
> i.Divided_hh_income i.New_past_sick i.New_chronic_disease

      Source |       SS       df       MS              Number of obs =     172
-------------+------------------------------           F(  8,   163) =    5.34
       Model |  7.27825804     8  .909782255           Prob > F      =  0.0000
    Residual |  27.7624396   163  .170321716           R-squared     =  0.2077
-------------+------------------------------           Adj R-squared =  0.1688
       Total |  35.0406977   171  .204916361           Root MSE      =   .4127

---------------------------------------------------------------------------------------------------------------
                                 Reg_no_admit |      Coef.   Std. Err.      t    P>|t|     [95% Conf. Interval]
----------------------------------------------+----------------------------------------------------------------
                                  New_country |
                                        ARAB  |    .486319    .101525     4.79   0.000     .2858454    .6867927
                                      OTHERS  |   .1115636   .0708073     1.58   0.117    -.0282543    .2513814
                                              |
                                      New_sex |
                                      Female  |   .0437014   .0646008     0.68   0.500    -.0838609    .1712637
                                              |
                                      New_age |
More than 15 years but not over than 60 years |   .0083513   .0680394     0.12   0.902     -.126001    .1427036
                           Over than 60 years |   -.146606   .2123527    -0.69   0.491    -.5659229    .2727109
                                              |
                            Divided_hh_income |
                               Income<=45707  |   -.039988   .1018335    -0.39   0.695    -.2410709    .1610949
                                              |
                                New_past_sick |
                                         Yes  |   .1746409   .0693757     2.52   0.013       .03765    .3116318
                                              |
                          New_chronic_disease |
                                         Yes  |   .1998674   .0893358     2.24   0.027     .0234628    .3762719
                                        _cons |   .0126931   .1112152     0.11   0.909    -.2069151    .2323013
---------------------------------------------------------------------------------------------------------------

. **Predict the probability of linear model**
. predict predLPM
(option xb assumed; fitted values)
(5 missing values generated)

. summ predLPM

    Variable |       Obs        Mean    Std. Dev.       Min        Max
-------------+--------------------------------------------------------
     predLPM |       178    .2861857    .2044262  -.0272949   .8855851

.
end of do-file
```

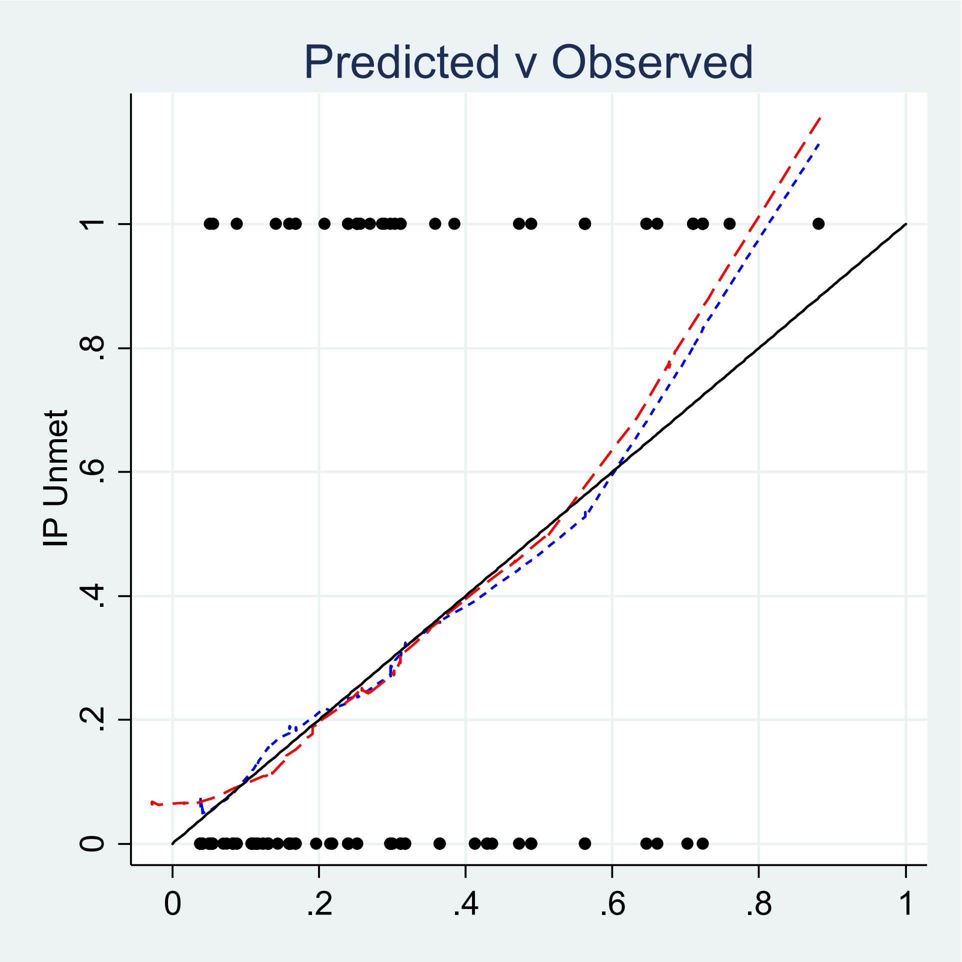
Predicted v Observed
IP Unmet
0
.2
.4
.6
.8
1
0
.2
.4
.6
.8
1

```
. **Check Statistical tests of Fit for IP by using Hosmer-Lemeshow Statistic(H-L)**
. quietly logistic Reg_no_admit i.New_country i.New_sex i.New_past_sick ///
> i.New_chronic_disease

. estat gof, group(8)

Logistic model for Reg_no_admit, goodness-of-fit test

  (Table collapsed on quantiles of estimated probabilities)

       number of observations =       172
             number of groups =         8
      Hosmer-Lemeshow chi2(6) =         7.84
                  Prob > chi2 =         0.2498

. estat gof, group(10)

Logistic model for Reg_no_admit, goodness-of-fit test

  (Table collapsed on quantiles of estimated probabilities)
  (There are only 9 distinct quantiles because of ties)

       number of observations =       172
             number of groups =         9
      Hosmer-Lemeshow chi2(7) =        10.79
                  Prob > chi2 =         0.1481

. estat gof, group(20)

Logistic model for Reg_no_admit, goodness-of-fit test

  (Table collapsed on quantiles of estimated probabilities)
  (There are only 14 distinct quantiles because of ties)

       number of observations =       172
             number of groups =        14
     Hosmer-Lemeshow chi2(12) =        13.23
                  Prob > chi2 =         0.3529

. estat gof, group(30)

Logistic model for Reg_no_admit, goodness-of-fit test

  (Table collapsed on quantiles of estimated probabilities)
  (There are only 16 distinct quantiles because of ties)

       number of observations =       172
             number of groups =        16
     Hosmer-Lemeshow chi2(14) =        15.23
                  Prob > chi2 =         0.3627

.
end of do-file
```

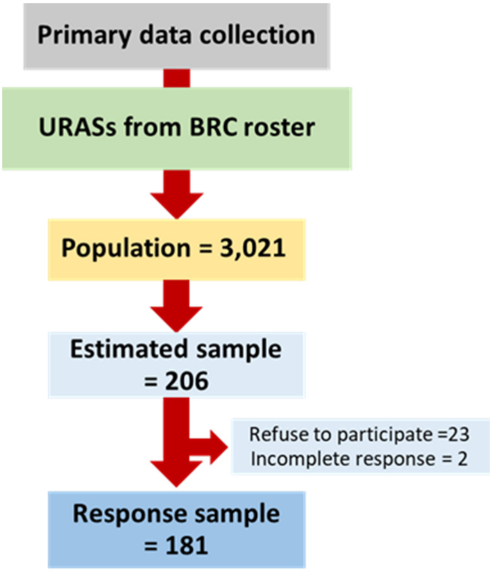

Figure 1. Participant recruitment process
